# Coffee and Risk of Pancreatic Cancer: Insights from Two-Sample and Multivariable Mendelian Randomization Analyses

**DOI:** 10.3390/nu16213723

**Published:** 2024-10-31

**Authors:** Yin Lu, Peng Wang, Haiyan Liu, Tiandong Li, Han Wang, Donglin Jiang, Ling Liu, Hua Ye

**Affiliations:** 1 College of Public Health, Zhengzhou University, Gaoxin District, Zhengzhou 450001, China; luyinsimple@163.com (Y.L.); zzdxlhy@163.com (H.L.); litiandong@outlook.com (T.L.); dionysian1996@foxmail.com (H.W.); jdl4961@163.com (D.J.); liulingchangzhi123@163.com (L.L.); 2 Henan Key Laboratory of Tumor Epidemiology and State Key Laboratory of Esophageal Cancer Prevention and Treatment, Zhengzhou University, Zhengzhou 450001, China; wangpeng1658@hotmail.com

**Keywords:** coffee, pancreatic cancer, Mendelian randomization, GWAS, epidemiology

## Abstract

Background: The association between coffee and pancreatic cancer risk has reported inconsistent results. Therefore, a Mendelian randomization (MR) study was undertaken to investigate the association between coffee and pancreatic cancer from a genetic perspective. Methods: In East Asian and European populations, independent genetic variants strongly associated with coffee were chosen as instrumental variables (IVs) from relevant genome-wide association studies (GWASs). GWAS data for pancreatic cancer were obtained from the JENGER (Japanese Encyclopedia of Genetic Associations by Riken) project and GWAS catalog database. Two-sample (TSMR) and multivariable Mendelian randomization (MVMR) analyses were conducted to investigate the genetically predicted causal relationship between coffee consumption and pancreatic cancer. A fixed-effect meta-analysis was employed to aggregate estimates from the two populations to reveal the overall association. Results: Both in East Asian and European populations, an increase in coffee intake of a cup per day was not associated with pancreatic cancer risk, regardless of coffee type (including caffeine drinks, instant coffee, decaffeinated coffee, ground coffee, etc.). The results aligned with the findings of the meta-analysis (OR = 1.100, 95%CI = 0.862–1.403, *p* = 0.450). Also, for coffee intake with positive results in the TSMR analysis (OR = 1.739, 95%CI 1.104–2.739, *p* = 0.017), consistent negative results were observed after adjusting for potential confounders (smoking traits, drinking, type 2 diabetes, body mass index) in the MVMR analyses. Conclusions: This study found no genetically predicted causal relationship between coffee consumption and pancreatic cancer risk.

## 1. Introduction

The American Cancer Society (ACS) reports that pancreatic cancer ranks as the third most common cause of cancer-related death for both men and women [1]. It often presents without symptoms in its early stages, and the 5-year relative survival rate for advanced cases is just 3% [2], accounting for almost as many deaths as cases [3]. At present, relatively few indicators have been identified for the early diagnosis of pancreatic cancer, and their sensitivity and specificity are relatively limited. Currently, widespread population-based screening for pancreatic cancer is not considered a practical option [4]. Therefore, the identification of risk factors for primary prevention has important public health implications, and can reduce the incidence and consequences of the disease to a certain extent.

Known risk factors for pancreatic cancer include body mass index (BMI), diabetes, cigarette smoking, drinking, pancreatitis, and so on [5]. A case–control study published in *The New England Journal of Medicine* [6] in 1981 found a positive relationship between coffee and pancreatic cancer. In the subsequent 40 years, high-quality meta-analyses [7] and prospective studies [8,9,10,11] have reported inconsistent results. Alternatively, the findings of Cynthia Morton et al. [12] showed that coffee consumption was associated with a reduced risk of alcohol-related pancreatitis. La Vecchia C et al. [13] found the relative risk of pancreatic cancer to be 1.2 for moderate and 1.4 for heavy drinkers, and they considered that this modest residual association was at least partially associated with smoking. In general, observational studies related to diet tend to interference by other diet and lifestyle factors. Hence, due to the possibility of residual confounding in relation to the above known risk factors and other sources of bias, we doubt that a cause-and-effect relationship between coffee and the risk of pancreatic cancer has been sufficiently established.

Mendelian randomization (MR) is a method that involves using genetic variants or modifiable exposures associated with biomarkers to investigate the causal effects of these factors on disease outcomes [14]. The MR design can strengthen causal inference [15] and reduce the impact of residual confounding and reverse causation [16]. Building on the strength of MR analysis, this study employed MR to reveal the causal effect between coffee and pancreatic cancer risk.

## 2. Methods

### 2.1. Study Design

**Figure 1:**
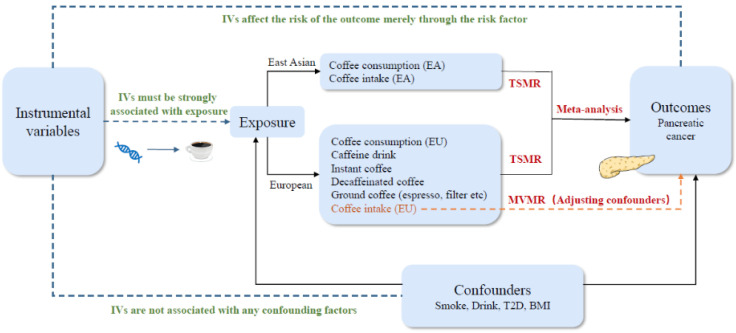
Flowchart of the study design. Abbreviations: EA, East Asian; EU, European; IVs, instrumental variables; TSMR, two-sample Mendelian randomization; MVMR, multivariable Mendelian randomization; T2D, type 2 diabetes; BMI, body mass index.

MR analysis employs single-nucleotide polymorphisms (SNPs) as instrumental variables (IVs) to determine causalities [17]. The IVs need to satisfy three assumptions: (a) IVs must be strongly correlated with exposure factors. (b) The IVs must be independent of any known confounders. (c) The IVs are not associated with an outcome and can affect the risk of outcome only through exposure, not via any other pathways. Figure 1 shows the study design. We conducted the TSMR analyses using GWAS data from East Asian and European populations to examine the genetically predicted causal relationship between coffee consumption/intake and pancreatic cancer risk. A meta-analysis was performed to combine the estimates of coffee consumption/intake in the two populations. An MVMR analysis was employed to reveal the direct causal effect between coffee intake and pancreatic cancer, while accounting for possible confounding factors.

### 2.2. GWASs for Coffee and Other Traits

A phenotype can be a disease, outcome, or trait, and GWASs can tell us whether a genetic variant is associated with a phenotype. This study obtained eight forms of GWAS data associated with coffee phenotypes, which came from the GWAS catalog, IEU OpenGWAS project, Nealelab, and Yanglab. Coffee consumption/intake was assessed using self-reported questionnaires and included the daily intake of decaffeinated coffee, instant coffee, ground coffee, and other types of coffee. GWAS summary statistics for drinking and smoking traits, including drinks per week, status of smoking initiation, age of initiation, cigarettes per day, and smoking cessation, were obtained from the Sequencing Consortium of Alcohol and Nicotine Use (GSCAN) project [18]. The smoking-related traits outlined above reflect various stages of tobacco use and addiction, such as starting smoking, developing regular use, the quantity smoked, and quitting. GWAS summary data for BMI were sourced from the Genetic Investigation of Anthropometric Traits (GIANT) consortium [19]. Furthermore, summary data on the genetic association of type 2 diabetes (T2D) were obtained from R10 data published in the FINNGEN study. Details of the phenotypes included in the study are provided in Table S1.

### 2.3. GWASs for Pancreatic Cancer

The GWAS summary statistics for pancreatic cancer were derived from the Japanese Encyclopedia of Genetic Associations by Riken (JENGER) project and the open access GWAS catalog database. JENGER is a comprehensive project that covers a wide range of genetic associations, including those related to diseases, traits, and drug responses, while the GWAS catalog is a comprehensive and publicly accessible database that contains information about GWASs. The JENGER project provided GWAS summary data for pancreatic cancer in East Asian populations (442 cases and 195,745 controls) [20]. This population was mainly from Japan, and the male-to-female ratio was 1.87:1 in the case group and 1:1 in the control group. The mean age was 64. The summary GWAS data for pancreatic cancer in the European population consists of two parts: UK Biobank (471 cases and 359,825 controls; mean age was 65.9) and Kaiser Permanente Genetic Epidemiology Research on Adult Health and Aging (GERA) cohorts (192 cases and 50,525 controls; mean age was 76) [21]. Details are provided in Table S1. Furthermore, according to ICD-10, pancreatic cancer is defined as C25.

### 2.4. Selection Criteria for Genetic Instruments

Genetic instruments for coffee consumption (EA), coffee intake (EU), ground coffee, smoking and drinking traits, BMI, and T2D were all selected under genome-wide significance thresholds (*p * < 5 × 10^−8^). To include more SNPs that contributed to other coffee-related phenotypes, a more relaxed threshold (*p* < 5 × 10^−6^) was used; this had been used previously in many other MR studies [22,23]. To reduce the effect of linkage disequilibrium (LD) on the results of random allelic isolation, strict conditions were set (LD threshold of *r*^2^  <  0.001 and 10,000 kilobase apart from each other). Since a particular SNP may be associated with multiple phenotypes, that is, genetic variants influence outcomes through other risk factor pathways, the PhenoScanner v2 was used to remove SNPSs associated with potential confounders (Table S2). This study offers two approaches for calculating the F-statistics in TSMR analysis, with a value exceeding 10 indicating a robust instrumental variable. The calculation method is as follows:

(a) The *F* statistic was calculated as the square of the exposure with genetic association, divided by the square of its standard deviation: $F=\frac{{BETA}^{2}}{SE^{2}}$ [24,25].
(b) The total *F*-statistic for each IV was calculated according to the following formula: $F=(\frac{R^{2}}{1-R^{2}})(\frac{N-K-1}{K})$ [24]. In this formula, *N* and *K* represent the number of exposures (sample size) and the number of IVs, respectively, and *R*^2^ denotes the total explanation of the exposure variance. So, when *K* = 1, the strength of individual SNPSs was measured by calculating the *F*-statistic: $F=\left(\frac{R^{2}}{1-R^{2}}\right)\left(N-2\right)$ [26]. The *R*^2^ was calculated by the following formula: $R^{2}=\frac{2EAF(1-EAF){BETA}^{2}}{\left(2EAF\left(1-EAF\right){BETA}^{2}\right)+(2EAF\left(1-EAF\right)N{SE}^{2})}$ [26,27]. *EAF* denotes the frequency of the effect allele, *BETA* represents the estimated genetic impact on the exposure, and *SE* indicates the standard error of the genetic effect. Furthermore, in the MVMR analysis, the *F*-statistic was measured by the ‘MVMR’ R package. Additionally, for SNPSs with missing effect allele frequencies, we used an open-source algorithm called ‘snp_add_eaf’, “https://github.com/linjf15/MR_tricks/blob/main/GWAS_preprocessing/snp_add_eaf.R (accessed on 17 October 2024)” to interpolate.

### 2.5. Statistical Analysis

#### 2.5.1. MR Analysis and Meta-Analysis

The inverse variance weighting (IVW) method was used as the primary approach in all MR analyses, which delivers a weighted regression for causal estimates tailored to instrumental variables, ensuring stable causal inference even when horizontal pleiotropy is present [28]. In the univariate TSMR analysis, the weighted median and MR-Egger estimation were also employed to evaluate the reliability of the IVW results. If at least half of the total weight is attributed to valid IVs, and the weighted median model can yield dependable estimates [29]. The MR-Egger method can still provide estimates after correcting for pleiotropy [30]. Moreover, a fixed-effects meta-analysis was used to combine the TSMR estimates of coffee consumption/intake across different populations. To reveal the direct effect of an extra cup of coffee per day on pancreatic cancer risk, a multivariate MR study was conducted, which can simultaneously detect the causal relationships of multiple risk factors [31]. In addition to the three analysis methods used by TSMR above, the Least Absolute Shrinkage and Selection Operator (LASSO) method was added to the MVMR analysis. LASSO regression penalizes parameters that contribute little to model fit, simultaneously performing variable selection and shrinkage [32].

#### 2.5.2. Sensitivity Analyses

Sensitivity analyses were performed via the IVW, MR-PRESSO, MR-Egger, and LASSO regression methods. The heterogeneity of the results was evaluated by Cochran’s Q-test from the IVW method. MR-Egger regression allows correction for a pleiotropy of SNPs; the intercept served as a measure for horizontal pleiotropy (horizontal pleiotropy was observed with *p* < 0.05) [30]. The Weighted Median Estimator method is more robust for pleiotropy because it does not rely on all SNPs being valid instrumental variables. The MR-PRESSO method identifies outliers among SNPs with pleiotropic effects and provides estimates after excluding these outliers [33]. In MVMR analysis, LASSO regression is used to solve the multicollinearity between variables and identify SNPs with poor correlation. The power calculations were conducted, considering that genetic variants explain only a fraction of the phenotypic variation. An online website tool “https://shiny.cnsgenomics.com/mRnd/ (accessed on 17 October 2024)” was utilized to measure the statistical power of MR analysis (Table S3). For MR analyses, statistical power greater than 0.75 is considered a convincing result. In this study, the main TSMR analysis used to measure coffee consumption and pancreatic cancer risk had a statistical power greater than 78%, with a type I error rate of 0.05.

MR analyses and sensitivity analyses were performed in R (version 4.3.1) with R packages ‘TwoSampleMR’, ‘MRPRESSO’, ‘MendelianRandomization’, and ‘MVMR’.

### 2.6. Ethics Approval

All GWAS datasets used in this study are publicly available without any access restrictions. All studies had been approved by a relevant ethical review board and participants had given informed consent.

## 3. Results

### 3.1. Genetic Instruments

According to the selection criteria of the genetic instruments, after removing 5 SNPs that might be associated with BMI, T2D, and drinking (Table S2), a total of 21 and 84 independent SNPs were selected as IVs for coffee in East Asian and European populations, respectively. The number of SNPs included in different phenotypes, the proportion of variance explained for each phenotype, and significance thresholds for the selection of IVs are shown in Table 1. Details of all SNPs included in the TSMR and MVMR analyses are provided in Tables S4–S11. In the univariate TSMR analysis, two methods were provided to calculate the F-statistics of individual SNPs and the combined F-statistics of SNPs with different phenotypes. The results all showed that the F-statistics of the IVs used were ≥10, which suggested strong instrument variables (Table 1 and Table S4). Additionally, F-statistics were calculated for the instrumental variables after adjustment for potential confounders in the MVMR analysis (Table S12).

### 3.2. The TSMR Analysis (IVW) and Meta-Analysis

**Figure 2:**
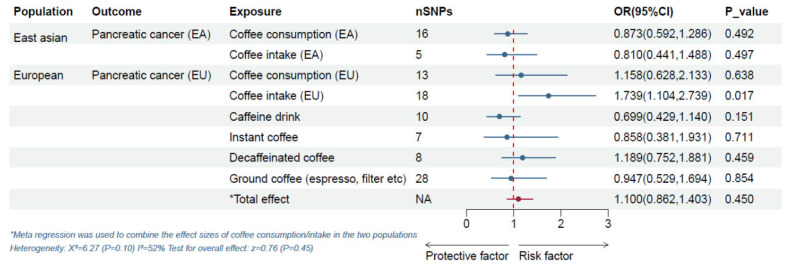
TSMR analysis and meta-analysis of relationship between an extra cup of coffee per day and pancreatic cancer risk. Notes: The reported values were calculated by the IVW method. Abbreviations: EA, East Asian; EU, European; SNP: single-nucleotide polymorphism; OR: odds ratio. CI: confidence interval; NA, not applicable.

Figure 2 shows the TSMR analysis and meta-analysis of an extra cup of coffee per day and pancreatic cancer risk. In the East Asian population, there was no statistical association between an additional cup of coffee per day and pancreatic cancer risk (*p* = 0.492 and 0.497, respectively). Similarly, in the European population, most TSMR analyses do not support an association between an extra cup of coffee per day and pancreatic cancer risk, regardless of coffee type (including caffeine drinks, instant coffee, decaffeinated coffee, ground coffee, etc.). However, an independent TSMR analysis (‘Coffee intake (EU)’, one of the coffee phenotypes) found that coffee was associated with an increased risk of pancreatic cancer (IVW: OR = 1.739, 95%CI = 1.104–2.739, *p* = 0.017). But this statistical association became insignificant when the other two methods of analysis were used (MR-Egger: OR = 1.808, 95%CI 0.736–4.441, *p* = 0.215; weighted median: OR = 1.409, 95%CI = 0.767–2.591, *p* = 0.269), as shown in Table S13. Also, estimates of coffee consumption/intake in East Asian and European populations were combined using a fixed-effects meta-analysis, and the results showed no statistically significant association between an extra cup of coffee per day and pancreatic cancer risk (OR = 1.100, 95%CI = 0.862–1.403, *p* = 0.450; *p* for heterogeneity = 0.10, I^2^ = 52%).

The results remained consistent across the sensitivity analyses (Table 2). No notable heterogeneity was found in any of the TSMR analyses (Cochran’s Q-test *p* > 0.05). There was no evidence for a significant intercept (Egger intercept *p* > 0.05), and the global test was insignificant (*p* > 0.05), indicating that there was no horizontal pleiotropy observed. The MR-PRESSO method did not detect outliers that needed to be corrected, meaning that the association of the negative results is robust.

### 3.3. MVMR Analyses: Adjusting for Potential Confounders

**Figure 3:**
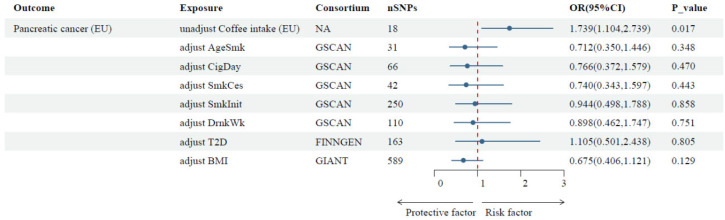
The causal effect of coffee intake on pancreatic cancer, adjusting for smoking traits, drinking, T2D, and BMI. Notes: The reported values were calculated by the IVW method. Abbreviations: AgeSmk, age of initiation; CigDay, cigarettes per day; SmkCes, smoking cessation; SmkInit, smoking initiation; DrnkWk, drinks per week; T2D, type 2 diabetes; BMI, body mass index; NA, not applicable.

For the positive results for ‘coffee intake’ in the TSMR analysis, an MVMR analysis was conducted adjusting for potential confounders, which showed that after adjusting for seven confounders (including drinking, four smoking traits, T2D, and BMI), no causal association was observed between an extra cup of coffee per day and pancreatic cancer risk (Figure 3). The causal relationship of the negative results remained robust even after using the other three methods of analysis (MR-Egger, Median, and LASSO), as shown in Table S14.

Sensitivity analyses using MR-Egger regression in the MVMR analysis showed no evidence of horizontal pleiotropy, and heterogeneity was detected only in MVMR analyses adjusted for T2D. Even so, the application of IVW random-effects methods and the absence of horizontal pleiotropy indicate that our findings are probably not influenced by heterogeneity. Moreover, the results of the LASSO analysis showed that 96–100% of the genetic instruments used in the MVMR analysis were valid (Table 3).

## 4. Discussion

In this study, TSMR analysis was used to comprehensively evaluate whether there is a causal relationship between an extra cup of coffee per day and pancreatic cancer risk, based on East Asian and European populations. Most of the results showed no clear evidence to support a causal relationship between coffee and pancreatic cancer risk, and the meta-analysis also showed no causal relationship. For the only positive result in the TSMR analysis, we adjusted for potential confounders (smoking traits, drinking, T2D, and BMI) using MVMR analysis, which also showed a negative result.

In previous observational studies [9,10] and their corresponding meta-analyses [7,34], the relationship between coffee consumption and pancreatic cancer has been contradictory. Many observational studies have reported a positive association between coffee consumption and pancreatic cancer risk. However, it is often difficult for these studies to completely eliminate the influence of confounding factors, especially those related to lifestyle and dietary patterns, such as smoking and alcohol consumption, which may be the real cause of increased pancreatic cancer risk, rather than coffee itself. Unlike previous population-based observational studies, this study is the first to use Mendelian randomization to reveal the link between coffee and pancreatic cancer risk from the perspective of genetic variation. Since genetic variants are unmodifiable and not affected by disease status, the MR design can reduce confounding and reverse causality. In addition, the study adjusted for several additional confounders associated with pancreatic cancer, enhancing the robustness of the results.

In addition to confounding factors that are difficult to eliminate, the inconsistent results of these studies may also be related to the mechanism of action of other components in coffee. A recent study [35] showed that caffeine may alleviate disease progression through Ca^2+^ regulation and gut–pancreatic axis regulation, but other ingredients in coffee drinks may bias this protective effect. Additionally, caffeine, along with other compounds found in coffee or factors associated with coffee consumption, may modulate K-Ras activation in exocrine pancreatic cancer by disrupting DNA repair mechanisms, cell-cycle checkpoints, and apoptosis. Porta M et al. [36] reported that pancreatic cancer patients with a K-Ras mutation drink more coffee than those without the mutation. However, the research found no overall relationship between coffee drinking and pancreatic cancer; this paradox could be explained by the possibility that coffee drinking raises the risk of pancreatic cancer associated with K-Ras mutations, while simultaneously lowering the risk of cancer in individuals without these mutations (the “wild type”). Thus, there may be no overall relationship [37]. On the other hand, as a result of coffee roasting processes and origin factors, in addition to micronutrients such as iron, zinc, manganese and copper, coffee can also contain toxic metals such as lead, chromium, and cadmium [38]. These toxic metals are closely related to the body’s antioxidant defense system and DNA repair. Zinc and copper are involved in the maintenance of REDOX homeostasis, and imbalance in the zinc/copper ratio is related to the risk of cancer [39]. Cadmium and chromium metals can induce oxidative stress and DNA repair defects [40], and molybdenum exposure affects the risk of pancreatic cancer by disrupting the metabolic processes associated with molybdenum-containing enzymes [41]. These components associated with coffee consumption are easy to overlook; while coffee is not considered a risk factor for the primary prevention of pancreatic cancer from our findings, excessive coffee consumption still requires caution to prevent potentially ignoring other factors lurking around that are attributable to a substantial number of pancreatic cancer cases.

In the MR analysis of the validity of the results, the main focus is on pleiotropy, especially horizontal pleiotropy, which implies that genetic variants influence pancreatic cancer risk not through coffee, but through other pathways. In this study, at first, a PhenoScanner was used to identify and remove SNPs that might be associated with other pleiotropic pathways. Second, MR-Egger regression, weighted median, and MR-PRESSO methods were used to address pleiotropy; the three methods were consistent in their conclusions, and although the estimates generated by the different methods may have differed slightly, none of them showed substantial pleiotropy, which indicates the robustness of our results across methods. Moreover, even after adjusting for some known confounders in the MVMR, there may still be confounders that are not identified or cannot be measured. These factors may influence both exposure and outcomes, resulting in a bias in causal inference. However, no evidence of horizontal pleiotropy was found in the study, suggesting that the observed causal estimates were less influenced by confounding factors.

This study has several strengths. The key advantage is the MR design, which significantly reduced the impact of confounding and reverse causality [15]. Secondly, due to variations in caffeine content and other components across different types of coffee [42], the associations with pancreatic cancer may differ depending on the type of coffee consumed. Therefore, the present study used multiple coffee-related exposure phenotypes (such as instant coffee, decaffeinated coffee, and ground coffee) for causal estimation in East Asian and European populations to ensure consistency and thus to obtain credible causal inferences. In addition, multivariate MR analysis was used to adjust for residual confounding factors to strengthen the causal inference between coffee and pancreatic cancer risk. The study population was limited to European and East Asian populations, and the studies were independent of each other, which effectively reduced the bias caused by the population structure.

There are some limitations to this study. To minimize bias from sample overlap, this study selected GWAS data from a variety of sources and populations whenever possible, but due to the large and concentrated sample size, there may still be potential sample overlap. Nonetheless, in the TSMR analysis, SNPs strongly associated with exposure were chosen, and all estimates had F-statistics greater than 10, indicating that any bias from partial sample overlap was likely minimal. Firstly, to include more SNPs, a more lenient threshold (*p* < 5 × 10^−6^) was used, which, while improving statistical power and the proportion of genetic variation explained, may have increased the risk of pleiotropy and biased the results. Therefore, the study used a more rigorous sensitivity analysis and controlled for possible pleiotropic pathways, and all SNPs had F-statistics greater than 10, making the association of instrumental variables with exposure strong to ensure reliable results. Secondly, differences in gene structure in different populations may influence the effects of genes related to coffee metabolism; for example, the frequency and effects of genetic polymorphisms associated with coffee consumption, such as the CYP1A2 [43] and AHR [44] genes, vary across ethnic populations. And coffee consumption patterns may vary depending on cultural, social, and economic factors, such as the production method of coffee, the use of additives, differences in consumption, etc. Due to the existence of genetic and environmental differences between different populations, replication studies in populations with different ethnic and cultural backgrounds are still needed. Thirdly, the MR analysis could not be stratified by age, sex, or pancreatic cancer severity due to the limitations of the GWAS data. Due to the lack of more GWAS data on specific coffee-dose stratification, linear or non-linear trends between coffee intake and pancreatic cancer risk could not be analyzed, which also provides a direction for follow-up research. Finally, there were limitations to the statistical method; MR analyses rely on GWAS data to identify genetic variants associated with exposure, and results may be biased if GWAS studies are underpowered. In addition, MR assumes that there is no interaction between genetic variants and environmental factors, which may deviate from the true causal link if environmental factors can regulate the effect of genetic variants on exposure or outcomes [45]. Therefore, our study provides supporting evidence and recommendations primarily from a genetic perspective, and we encourage additional prospective studies to enhance true causal associations.

Future research directions should pay attention to the following points. First, if quantitative GWAS data on coffee consumption are available, it is necessary to assess the linear or non-linear association between coffee consumption and pancreatic cancer risk. Second, it is necessary to include more populations from different regions to complement this study so that the conclusions are universally applicable. Lastly, different components of coffee may act through different pathways, and an integrated analysis of the effects of these components may help explain why no clear causal relationship has been found.

## 5. Conclusions

In conclusion, our findings provide genetic evidence suggesting that there is no causal relationship between coffee and pancreatic cancer risk.

## Figures and Tables

**Table 1:** Summary of each genome-wide association study in the TSMR analysis.

Phenotype	Sample Size	No. of SNPs	*r* ^2^	*F*	Significant Threshold
East Asian					
Coffee consumption (EA)	152,634	16	0.9%	87.612	5 × 10^−8^
Coffee intake (EA)	2338	5	5.1%	25.043	5 × 10^−6^
European					
Coffee consumption (EU)	64,001	13	0.5%	24.007	5 × 10^−6^
Coffee intake (EU)	263,464	18	0.7% *	97.550	5 × 10^−8^
Caffeine drink	418,744	10	0.1%	21.980	5 × 10^−6^
Instant coffee	64,001	7	0.3%	24.123	5 × 10^−6^
Decaffeinated coffee	64,001	8	0.3%	22.045	5 × 10^−6^
Ground coffee (espresso, filter, etc.)	283,449	28	0.3%	40.024	5 × 10^−8^

Abbreviations: TSMR: two-sample Mendelian randomization; SNP: single-nucleotide polymorphism; *r*^2^: the proportion of variance explained for each potential risk factor; significant threshold: genetic instruments (SNPs) for exposure were all selected at genome-wide significant threshold (*p* < 5 × 10^−8^ or *p* < 5 × 10^−6^); 0.7% *: the effect allele frequency for coffee intake (EU) was not available. Online tools were utilized to interpolate the effect allele frequency value to calculate the approximate estimation of *r*^2^.

**Table 2:** Sensitivity analysis of relationship between coffee and risk of pancreatic cancer in univariable TSMR analyses. Abbreviations: OR: odds ratio. CI: confidence interval. The OR and CI were calculated from the estimate and *p* value. NA: No outlier needs to be corrected.

Exposure	Heterogeneity		Pleiotropy		MR-Presso				
	Cochran’s Q-test		MR-Egger		Before Correction			Outlier Corrected	Global Test
	Q	*p* Value	Intercept	*p* Value	OR	95%CI	*p* Value		*p* Value
East Asian									
Coffee consumption (EA)	23.037	0.083	4 × 10^−4^	0.995	0.873	(0.476, 1.270)	0.503	NA	0.127
Coffee intake (EA)	1.445	0.836	0.024	0.869	0.810	(0.393, 1.227)	0.322	NA	0.869
European									
Coffee consumption (EU)	18.119	0.112	−0.011	0.894	1.158	(0.530, 1.785)	0.647	NA	0.118
Coffee intake (EU)	11.636	0.822	−0.003	0.922	1.739	(1.316, 2.161)	0.010	NA	0.805
Caffeine drink	9.008	0.437	0.052	0.488	0.699	(0.170, 1.229)	0.185	NA	0.442
Instant coffee	6.879	0.332	0.131	0.218	0.858	(0.009, 1.706)	0.723	NA	0.350
Decaffeinated coffee	4.589	0.710	−0.045	0.517	1.189	(0.794, 1.585)	0.391	NA	0.725
Ground coffee (espresso, filter, etc.)	23.718	0.646	0.041	0.607	0.947	(0.399, 1.494)	0.845	NA	0.651

**Table 3:** Sensitivity analysis of relationship between adjusted coffee intake and risk of pancreatic carcinoma in multivariable MR analyses.

Exposure	Heterogeneity		Pleiotropy		LASSO	
	Cochran Q Test		MR-Egger		NO. of Variants	NO. of Valid Instruments
	Q	*p* Value	Intercept	*p* Value		
Coffee intake adjusted by AgeSmk	28.460	0.493	0.019	0.322	31	31
Coffee intake adjusted by CigDay	71.945	0.232	0.025	0.201	66	65
Coffee intake adjusted by SmkCes	46.995	0.208	0.010	0.620	42	42
Coffee intake adjusted by SmkInit	243.386	0.571	0.027	0.096	250	250
Coffee intake adjusted by DrnkWk	106.780	0.516	−0.009	0.614	110	110
Coffee intake adjusted by T2D	193.269	0.042	−0.021	0.148	163	157
Coffee intake adjusted by BMI	606.377	0.281	0.017	0.111	589	587

Abbreviations: AgeSmk: age of initiation. CigDay: cigarettes per day. SmkCes: smoking cessation. SmkInit: smoking initiation. DrnkWk: drinks per week. T2D: type 2 diabetes. BMI: body mass index.

## Data Availability

All data used in this study are publicly available without any access restrictions. The data used in the analysis are presented in the Supplementary Materials.

## Supplementary Materials

The following supporting information can be downloaded at: https://www.mdpi.com/article/10.3390/nu16213723/s1, Table S1: The detail information of outcomes and exposure phenotypes included in this study; Table S2: Eliminating SNPs associated with known confounding factors; Table S3: Power calculations for Mendelian Randomization; Table S4: Single nucleotide polymorphisms used as instrumental variables in the analysis of TSMR; Table S5–S11: Instrumental variables of AgeSmk, CigDay, SmkCes, SmkInit, DrnkWk, T2D, BMI in the MVMR analyses of pancreatic cancer and coffee intake (EU); Table S12: F-statistics for all multivariable MR analyses; Table S13: TSMR analysis among coffee phenotypes and pancreatic cancer; Table S14: Results of multivariable Mendelian randomization adjusting potential confounders.
